# Editorial: The importance of the body-mind relationship in mental functioning and development of body-focused disorders in adolescence

**DOI:** 10.3389/fpubh.2023.1148279

**Published:** 2023-02-09

**Authors:** Stefania Cella, Paolo Cotrufo

**Affiliations:** Observatory on Eating Disorders, Department of Psychology, University of Campania “Luigi Vanvitelli”, Caserta, Italy

**Keywords:** body-focused disorders, anorexia, NSSI, self-image, tattooing, social insecurity

The interest in the body as a theater representing the psychic scene had changed. Bodies emaciated, obese, cut, burned, mutilated; hyper-tattooed bodies, pierced everywhere, naked or in disguise; Marvel superhero bodies, giants of muscles trained daily, surgically modified bodies, unrecognizable faces that end up resembling each other because they are children of the same plastic surgeon; physically young older people and the huge anti-aging cosmetics industry. The body that in the last 50 years has become the bearer of the symptom appears as a concrete body, no longer an expression of psychological symbolism, now seems to represent the subjectivity of those who inhabit it concretely. It seems that the body must be modified in symptoms and fashion trends to adapt to the needs of subjectification of individuals. The papers on body-focused disorders that you will find in this special issue probably refer to a further change in body-mind relation. After all, these are “problems” of the body which are nevertheless determined by people's conscious choices, especially adolescents. The voluntary malnutrition that we observe in anorexia, the binge eating and self-induced vomiting in bulimia, the self-cutting in non-suicidal self-injuries (NSSI), the choice of hyper-tattooing the body, together with the addiction to cosmetic surgery or body-building, seem to be the new ways to express a psychological discomfort through a sort of omnipotent control over one's body. The body turns out to be disciplined, it must submit to the will of a psychism and an idea of itself that finds self-expression in manipulating the body and its image. The problem of the appearance of our body seems to have become of extreme importance at different levels, it affects all of us.

This Research Topic focuses on the mind-body relationship, comprising a prospective study and seven empirical papers.

Wei et al. recruited 643 Chinese adolescents and found that depression mediates the relationship between stressful life events and non-suicidal self-injury (NSSI). Moreover, resilience was found to moderate such a pathway: the adverse impact of stressful life events on NSSI through depression was weaker in adolescents with higher levels of resilience. These findings echo the importance of the emotion regulation processes, suggesting new directions for intervention and prevention.

Kuo et al. utilized a longitudinal cohort of adolescents [i.e., Taiwan Youth Project (1)] to investigate the relationship between weight status and self-image. The cross-sectional analysis demonstrated that those with a higher BMI were more likely to report a lower level of self-image. Moreover, pubertal timing and athletic performance significantly mediate the pathway from weight status and self-image among females, whilst only athletic competence mediated such association within males. The longitudinal association showed that males' BMI and athletic competence at baseline significantly predicted self-image at 9-years follow-up, while only BMI at baseline was predictive of long-term self-image among females. Such a pattern of results suggests the crucial role of pubertal development in the mind-body linkage, highlighting the role of masculine and feminine sex-role ideals in constructing self-image.

Using a mixed-method design, Ojeda et al. collected data from 278 tattooed Mexican adults attending a free healthcare clinic in Tijuana's Zona Norte to assess tattoo removal motives among economically disadvantaged individuals. Overall, 69% of participants were interested in undergoing free tattoo removal services due to their appearance consequences, such as employment barriers, stigmatization, and discrimination. Also, having tattoos negatively impacted participants' mental health, contributing to feelings of discomfort and shame. Findings shed light on the body perception within a sample of structurally vulnerable adults and its impact on wellbeing, and social and work functioning.

Also, concerns about physical appearance have a negative impact on quality of life (QoL) among women with polycystic ovary syndrome (PCOS; *n* = 435). Barberis et al. showed the mediating role of dysmorphic concerns and eating disorder symptoms in the relationship between BMI and QoL. Therefore, such results highlight the potential importance of harmful relationships with one's own body and food, explaining why weight issues may be linked to different levels of QoL in PCOS individuals.

In their cross-sectional study, Ishikawa et al. used a large sample of adolescents (*n* = 9.998) to investigate the association between the subjective degree of influence (DOI) and thinness. Results showed that DOI was associated with the risk of being thin during adolescence, which is one of the main risk factors for eating disorders.

Using a network analysis approach, Jin et al. investigated the association between social anxiety disorder (SAD), appearance anxiety, and eating disorders (ED) in a sample of Chinese university students. Results demonstrated that appearance anxiety was associated with both ED and SAD, although showing different patterns of associations across genders. These findings provide preliminary evidence to guide the formation of body-positive interventions for young people.

Tang et al. carried out an in-depth study of adolescents' QoL. Findings demonstrated that negative life events were negatively related to QoL. However, resilience and social support weakened and reduced the adverse effects of negative life events on adolescents, mediating the pathway. Therefore, health intervention should address these factors during adolescence, as a period characterized by the tendency for increased exposure to adverse life events and a gradual decline in QoL satisfaction.

In the perspective study by D'Agostino et al., the authors emphasize the body's function for the adolescent as a primary means for the regulation of the self-other relationship and the development of a greater sense of self-agency. Although scientific studies have highlighted an increase in body disorders (e.g., non-suicidal self-injury and eating disorders) among adolescents during the pandemic, results should be interpreted with caution. Overestimating the associaTion between bodily disorders and the pandemic risks obscuring the complex network of factors involved in such relationships. Consequently, they concluded that there is a need to conceptualize such disturbances from a developmental psychopathological perspective.

We hope that the reader will find in this Research Topic a useful reference to a better and broader understanding of the importance of the body in mental functioning and body-focused disorders (i.e., eating disorders, non-suicidal self-injury).

## Author contributions

All authors listed have made a substantial, direct, and intellectual contribution to the work and approved it for publication.
